# COL2A1 and Caspase-3 as Promising Biomarkers for Osteoarthritis Prognosis in an *Equus asinus* Model

**DOI:** 10.3390/biom10030354

**Published:** 2020-02-26

**Authors:** Aya M. Yassin, Huda O. AbuBakr, Ahmed I. Abdelgalil, Marwa S. Khattab, Adel M. EL-Behairy, Eman M. Gouda

**Affiliations:** 1 Department of Biochemistry and Chemistry of Nutrition, Faculty of Veterinary Medicine, Cairo University, Giza 12211, Egypt; adelelbehairy@cu.edu.eg (A.M.E.-B.); eman_gouda@cu.edu.eg (E.M.G.); 2 Department of Surgery, Anesthesiology and Radiology, Faculty of Veterinary Medicine, Cairo University, Giza 12211, Egypt; ismael7591@cu.edu.eg; 3 Department of Pathology, Faculty of Veterinary Medicine, Cairo University, Giza 12211, Egypt; marwakhattab@cu.edu.eg

**Keywords:** osteoarthritis, donkey, matrix metalloproteinases, COL2A1, Caspase-3

## Abstract

Osteoarthritis (OA) is one of the most degenerative joint diseases in both human and veterinary medicine. The objective of the present study was the early diagnosis of OA in donkeys using a reliable grading of the disease based on clinical, chemical, and molecular alterations. OA was induced by intra-articular injection of 25 mg monoiodoacetate (MIA) as a single dose into the left radiocarpal joint of nine donkeys. Animals were clinically evaluated through the assessment of lameness score, radiographic, and ultrasonographic findings for seven months. Synovial fluid and cartilage samples were collected from both normal and diseased joints for the assessment of matrix metalloproteinases (MMPs) activity, COL2A1 protein expression level, and histopathological and immunohistochemical analysis of Caspase-3. Animals showed the highest lameness score post-induction after one week then decreased gradually with the progression of radiographical and ultrasonographic changes. MMP activity and COL2A1 and Caspase-3 expression increased, accompanied by articular cartilage degeneration and loss of proteoglycan. OA was successfully graded in Egyptian donkeys, with the promising use of COL2A1and Caspase-3 for prognosis. However, MMPs failed to discriminate between early and late grades of OA.

## 1. Introduction

Osteoarthritis (OA) is one of the most difficult chronic slowly progressive degenerative joint diseases in both human and veterinary medicine [1,2,3]. This disease mainly causes pain, lameness, and alterations in animal quality of life and welfare [4]. The equine industry attracts great attention, as the horse racing industry is worth over £3.45 billion to the UK economy and economic loss to this industry refers to diagnosed OA [5].

The etiology of OA is not well known, and its progression mainly alters articular cartilage homeostasis, which is avascular tissue with limited regenerative ability. The mechanical support and joint lubricant proprieties of articular cartilage are dependent on the integrity of its extracellular matrix (ECM) [6]. Cartilage ECM is rich in fibrillar collagens, especially type II, that occupy 80% of total collagen, large proteoglycans, and smaller hydrophilic macromolecules [7]. In normal physiological conditions, chondrocytes regulate the structural and functional integrity of cartilage through maintaining the equilibrium between the synthesis and degradation of the ECM components [8], while in OA, the cardinal surgical signs are characterized by progressive loss of articular cartilage, concurrently with reactive responses in the joint as subchondral osteoblast activation, osteophytes formation, and synovitis [9]. Additionally, histobiochemical analysis of OA attributes a change in proteoglycan metabolism and ECM loss that is easily evidenced by reduced Safranin-O staining [10].

The pathophysiology of OA is characterized by the launching of proinflammatory cytokines, such as interleukin-1β (IL-1β) and tumor necrosis factor-α (TNF-α); subsequently, cartilage degrades through two phases: the degradative and biosynthetic phase. In the degradative phase, proteolytic enzymes, including matrix metalloproteinases (MMPs) and the A Disintegrin and Metalloproteinase domain with Thrombospondin-like motifs family (ADAMTS), digest the ECM, accompanied by repairing the damaged cartilage in the biosynthetic phase [11,12]. However, matrix synthesis is rapidly inhibited by inflammatory factors through the down regulation of ECM genes with accelerating cartilage damage [13]. The major MMPs involved in the digestion of the ECM include the interstitial collagenases (MMP-1, MMP-8, MMP-13), gelatinases (MMP-2 and MMP-9), stromelysin 1 (MMP-3), and membrane-type collagenases (MMP-14 and MMP-16) [14]. In normal conditions, chondrocytes express MMP-1, MMP-8, and MMP-13 at low levels, whereas these enzymes were found to be expressed 10-fold higher in osteoarthritic joints, resulting in the digestion of cartilage collagen, which is considered a critical step in the loss of articular cartilage [15]. Recently, several studies have proved the correlation between cell death and matrix degradation of human and animal OA cartilage models [16,17,18], since matrix degradation results in the loss of survival mechanisms [19], calcification [20], and apoptosis [21]. Cartilage or chondrocyte apoptosis is mediated by a cascade of aspartate-specific cysteine proteases or several types of caspases, especially Caspase-3 which is one of the key mediators of apoptotic executioners in its execution phase [22].

Animal models are standard research tools for studying the pathogenesis, diagnosis, and powerful therapeutic intervention of different diseases [23,24]. The slowly progressive nature of OA necessitates certain criteria for the selection of appropriate animal models in OA studies, such as anatomical features of articular cartilage, long life, and the availability of collection of synovial fluid samples [25,26]. From all animal models, those which have articular cartilage closest to humans are equine species [27,28,29]. Several studies have been elucidated to study OA in horses, especially since they are good models for naturally occurring OA to rescue the horse racing industry and attract attention to human OA [30,31]. Donkey (*Equus asinus*) is one of the equine species and closest animal to the horse, making this species an alternative cheap animal model for studying equine diseases [32,33,34].

Experimental models of chemically induced OA using intra-articular injection of monoiodoacetate (MIA) were reported in rat [35], rabbit [36], pig [37], and horse [38,39].

The absence of real and effective treatment able to reverse the progressive changes of OA and the lack of adequate diagnostic tools brings out the need to develop early biomarkers for rapid medical intervention and achieve a good prognosis. Thus, the challenge of the present study is the early diagnosis of OA in donkeys using a reliable grading of the disease based on clinical, chemical, and molecular alterations. For achieving this purpose, OA in donkeys is induced chemically by MIA injection and the disease grading is followed up by X-ray and ultrasonography, monitoring the biochemical and molecular changes using the synovial fluid as a mirror for the joint environment.

## 2. Materials and Methods

### 2.1. Ethical Statement

This animal experiment followed the guidelines developed by the American Psychological Association (APA) for the ethical conduct of care and use of animals and approval was obtained from CU-IACUC with No. (CU/II/F/4/16).

### 2.2. Animals

Nine adult male donkeys aged 3–5 years, weighing 150–200 kg, of a healthy Egyptian local breed were subjected to comprehensive orthopedic examination, including an assessment of lameness during walking and trotting in straight lines and circles on hard ground [40,41]. The radiographical examination of the radiocarpal joints of both limbs and range of motion of joints (angle of flexion) were assessed to ensure that they were within normal limits and only animals proven healthy were selected for the present study.

### 2.3. Housing and Feeding

Animals were housed in an isolated and well-ventilated stable under standard environmental conditions (23 ± 1 °C, 55 ± 5% humidity, and 12 h light/dark cycle) at the Department of Surgery and anesthesiology, Faculty of Veterinary Medicine, Cairo University, Giza, Egypt. All donkeys were given free access to water and were given well-balanced rations. Donkeys were allowed to acclimatize for two weeks before the study. During the acclimatization period, animals were trained daily to familiarize them with the experimental conditions (investigators, environment, handling, vein puncture, and various outcome measures) and were walking daily for 15–20 min.

### 2.4. Study Design

#### 2.4.1. Control Sample Collection

At the beginning of the experiment, synovial fluid samples were collected from the left radiocarpal joint of all nine adult donkeys before osteoarthritis induction (day 0).

#### 2.4.2. Induction of Osteoarthritis

Under strict aseptic conditions, the left radiocarpal joint in each donkey was intra-articularly injected with 25 mg of MIA (sodium monoiodoacetate, Sigma-Aldrich, St. Louis, MO, USA) dissolved in 1 mL of sterile saline [34].

### 2.5. Clinical Assessment

#### 2.5.1. Evaluation of Lameness

Evaluation of lameness was done following the American Association of Equine Practitioners (AAEP) scale for lameness evaluation [23]: 0 = sound, 1 = lameness difficult to detect and inconsistent, 2 = lameness difficult to detect, but consistent, 3 = lameness consistently detectable on a straight line, and 4 = obvious lameness with marked head nodding [33].

#### 2.5.2. Radiographical Examination

X-ray films of the radiocarpal joints were taken weekly starting from day 0 (before MIA injection) to the end of the experiment (seven months). The radiographs were taken in dorso-palmar and latero-medial views. The settings of the X-ray machine were 50 KV, 3 mAs, and 75cm focal film distance. The degree of arthritic changes was scored according to the Osteoarthritis Research Society International (OARSI) classification of osteoarthritis of the knee [42].

#### 2.5.3. Ultrasonographic Procedures

The dorsal surface of the carpal region was clipped and shaved in all animals. Ultrasonographic examinations were performed using a Toshiba Just Vision 200 (Japan) machine equipped with 10 MHz linear probe. The probe was positioned along the length and width of the radio-carpal joint in the flexed position. Synovial fluid and articular cartilages were evaluated on day 0, one, two, three, five, and seven months after induction.

### 2.6. Experiments

#### 2.6.1. Sample Collection

##### Synovial Fluid Collection

Samples (about 1 mL) were collected from the left radiocarpal joint of each animal under complete aseptic conditions using a 23G needle before any interference at day 0, then weekly post-injection until the end of the study at seven months. Three donkeys were randomly euthanized at three, five, and seven months for cartilage sample collection. The collected samples were immediately centrifuged at 10,000× *g* for 20 min at 4 °C. The supernatants were aliquoted and stored at −20 °C for further analysis. Synovial fluid samples were diluted before use in a ratio (1:5) with 1× PBS (pH:7.2) to reduce the viscosity and measurement of total protein [43].

##### Cartilage Samples Collection

After euthanasia by intravenous injection of thiopental sodium (25 mg/kg) at three, five, and seven months, articular cartilage samples were collected from the left radiocarpal joint and right radiocarpal joint by transverse cut, then articular surfaces were macroscopically examined, followed by fixation in 10% neutral formalin buffer for histopathology and immunohistochemistry evaluation.

#### 2.6.2. Assessment of Matrix Metalloproteinases (MMPs) Enzymes Activity by Heparin-Enhanced Substrate Zymography

The total protein concentrations for diluted synovial fluid samples were measured by using the method described by Bradford, 1976 [44].

The activity of MMPs (collagenases; 1, 13 and gelatinases; 2, 9) was detected in 7.5% gelatin zymography by loading 10 µL heparin (0.3 mg/mL in 1× sample buffer without SDS) to lanes within 20–30 min after electrophoresis began [45].

#### 2.6.3. Western Blot Analysis

Detection of the COL2A1 protein by western blotting was carried out according to [46]. Briefly, equal amounts of total protein (30 µg) from different synovial fluid samples were loaded for each lane and separated by 10% SDS-PAGE, followed by electroblotting by tank transfer technique to the PVDF membrane. The primary antibody used was polyclonal anti-type II collagen, COL2A1 antibody (Chongqing Biospes, cat# YPA1669), and HRP-conjugate goat anti-rabbit IgG as a secondary antibody (Chongqing Biospes, cat#BSA1013). The desired specific bands were developed by using DAB horseradish peroxidase chromogenic kit (Chongqing Biospes, cat#BWR1069).

Both zymogram gels and western blot membranes were digitally scanned in the true color. The bands’ intensities were quantified using my Image analysis software v2.0 (Thermo scientific) after being conversed with the grayscale. For western blot analysis, COL2A1 blots were normalized to the total protein according to Fosang et al. [47].

#### 2.6.4. Histopathological Examination

Biopsies from cartilage were collected at different time intervals (three months, five months, and seven months from the start of the experiment) and fixed in 10% neutral formalin buffer. Fixed samples were then processed by the paraffin embedding technique, sectioned, and stained by hematoxylin and eosin stain and Safranin O. The stained tissue was examined by a light microscope and photographed by Olympus camera (XC30, Tokyo, Japan). The cartilage changes were graded according to the OARSI guidelines [48].

#### 2.6.5. Immunohistochemical Analysis

Caspase-3 was immunohistochemically stained in paraffin-embedded tissue sections. Briefly, after deparaffinization and rehydration, antigen retrieval was performed and the Caspase-3 primary antibody (Novus Biologicals, Centennial, CO, USA) was applied. The avidin-biotin-peroxidase complex method was then carried out according to the manufacturer’s protocol (Novus Biologicals) using diaminobenzidine as a substrate and hematoxylin as counterstain. The percentage area of positively stained tissue was measured using Image J software in three images/samples.

### 2.7. Statistical Analysis

The obtained data were statistically analyzed using the One-Way ANOVA Statistics, version 24.0 software (SPSS Inc., Chicago, IL, USA). Polynomial contrasts, post-hoc Duncan, and descriptive statistics were performed. The level of significance was set at *p* ≤ 0.05. The represented values are given as a standard error of the mean (SEM).

## 3. Results

### 3.1. Clinical Assessment

#### 3.1.1. Evaluation of Lameness

The animals provoked the highest lameness score (score 3) one-week post OA induction, then lameness decreased gradually with constant score (score1) from the second to the seventh month (Table 1).

#### 3.1.2. Radiographical Evaluation

**Figure 1:**
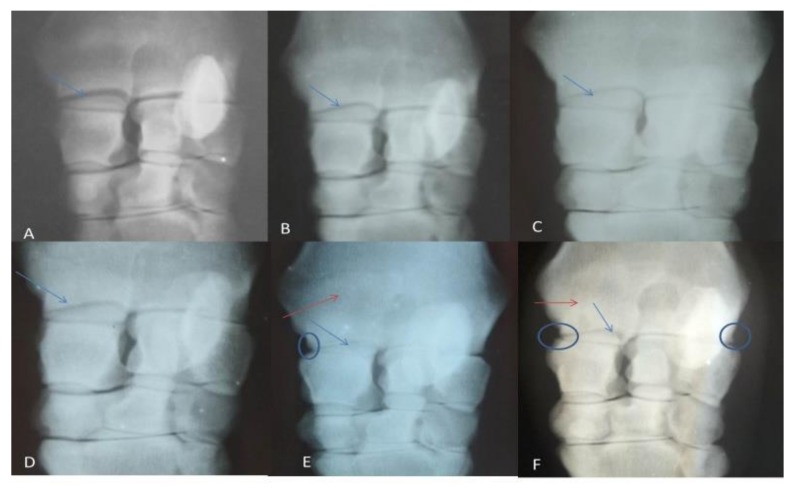
Showing radiographic grading: (**A**) normal (Day 0), (**B**) after one month of induction, (**C**) after 2 months, (**D**)after 3 months, (**E**)after 5 months, (**F**) After 7 months. The radio-carpal joint space showed gradual narrowing (blue arrows), subchondral bone sclerosis (red arrows) and osteophyte appeared in late stages (circle).

Radiographical findings revealed, according to OARSI:(1) joint space narrowing, which began to appear at the first month post-induction (grade 1), then gradually increased to be grade 2 at the second and third months, and reached grade 3 by the seventh month; (2) subchondral bone sclerosis, which began to appear from the third month post-injection, until the end of the experiment; (3) marginal osteophytes were observed in the fifth and seventh months, while bone attrition was not observed in the present study (Figure 1 and Table 2).

#### 3.1.3. Ultrasonographic Findings

**Figure 2:**
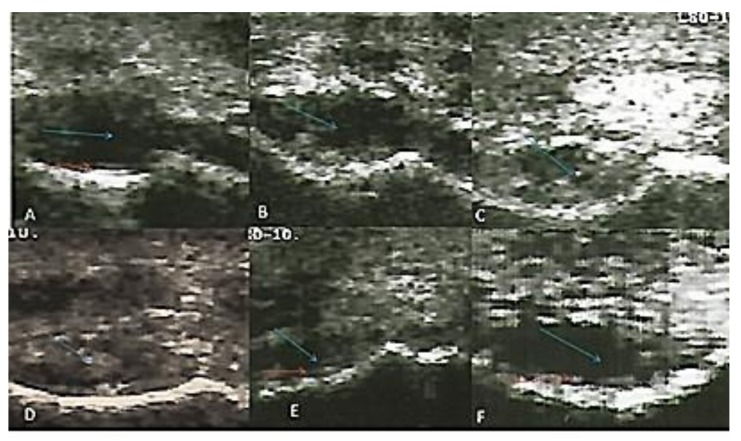
Transverse flexed scan of the radio-carpal joint at (**A**) day 0, (**B**) first month, (**C**) second month, (**D**) third month, (**E**) fifth month, (**F**) seventh month after induction. Synovial sac showed hypo-echoic mass within the synovial fluid (blue arrows). The articular cartilage appeared normal at day 0 and showed a defect in continuity seven months after induction (red arrows).

The radio-carpal joint at day 0 showed anechoic fluid, smooth articular cartilage, and regular bony alignment. The articular cartilage appeared as two thin echogenic lines above the hyper-echoic surface of the radio-carpal bone. The synovial sac showed that hypo-echoic mass within the synovial fluid was appeared after one month of induction and increased gradually until the third month; this mass decreased in the fifth and seventh months. Articular cartilage showed decreased echogenicity with a defect in its continuity at the seventh month after induction (Figure 2).

### 3.2. Assessment of Matrixmetalloproteinase Activity

**Figure 3:**
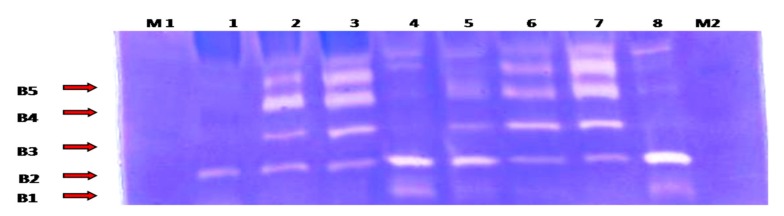
Heparin-enhanced gelatin zymography for MMP activity detection from the synovial fluid samples. Lane 1: represents day 0 (control) samples; lane 2: first week samples; lane 3: first month samples; lane 4: second month samples; lane5: third month samples; lanes 6 and 7: fifth month samples; and lane 8: seventh month samples. B1: corresponding to active MMP13 (48 KDa); B2: active MMP-1(57 KDa); B3: active MMP-2 (66 kDa); B4: active MMP-9 (86 KDa); and B5: latent MMP-9 (92 KDa). M1: pre-stained protein marker (10–175 KDa) (M1 was loaded at the beginning of the electrophoresis, while M2 was loaded 20min after the electrophoresis began).

Collagenase (MMP-1 and MMP-13) and gelatinase (MMP-2 and MMP-9) activities were detected by heparin-enhanced substrate zymography (Figure 3).

**Figure 4:**
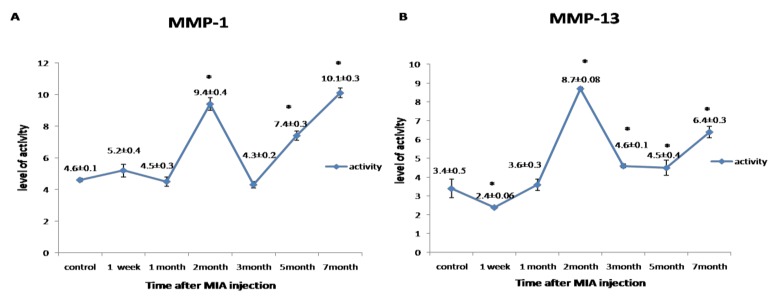
Graphical representation of the changes in the activity of matrix metalloproteinases (MMPs) during the disease progression post-monoiodoacetate (MIA) injection at day 0, 1st week, 1st month, 2nd month, 3rd month, 5th month, and 7th month. (**A**) Representing the activity of MMP-1. (**B**) Representing the activity of MMP-13. All data are represented as (mean ± SEM) for triplicate samples (*n* = 3). * denotes a significant difference from control samples at day 0 at *p* < 0.05.

Collagenase activity for MMP-1 and MMP-13 was found to be expressed in an undulating manner with two peaks of maximum expression detected in the second month and at the seventh month (*p* < 0.05) (Figure 4).

**Figure 5:**
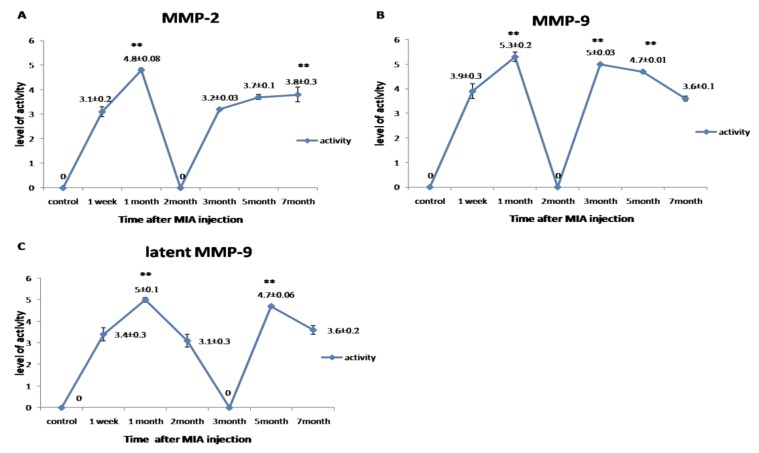
Graphical representation for the changes in the activity of MMPs during the disease progression post-MIA injection at day 0, 1st week, 1st month, 2nd month, 3rd month, 5th month, and 7th month. (**A**) Representing the activity of MMP-2. (**B**) Representing the activity of MMP-9. (**C**) Representing the activity of latent MMP-9. All data are represented as mean ± SEM for triplicate samples (*n* = 3). ** denotes significant difference from samples in the 1st week at *p* < 0.05.

Gelatinase activity for MMP-2 and MMP-9 was detected in a biphasic expression pattern; the first phase started from the first week to the first month, which recorded the highest value of its activity. The second phase started from the third to the seventh month. Meanwhile, the latent MMP-9 first phase of expression started from the first week to the second month, and the second phase began from the fifth month to the end of the experiment. The highest level of gelatinases activity was observed in the first month (*p* < 0.05) (Figure 5).

### 3.3. Immunoblot Analysis for COL2A1 Protein

**Figure 6:**
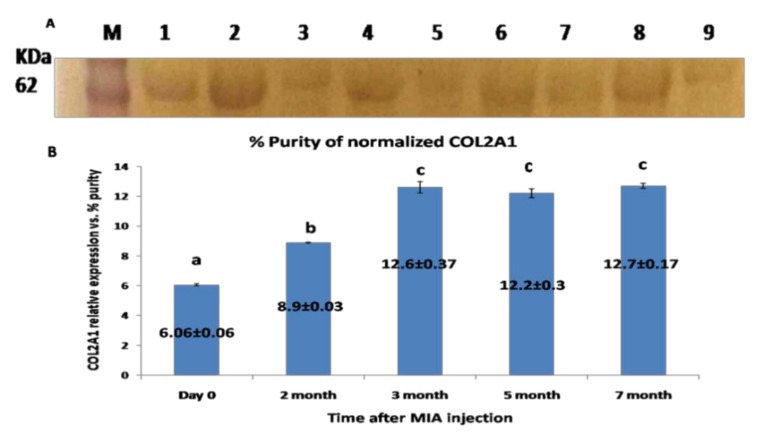
(**A**) Western blot membrane representing the specific band for COL2A1 protein from the synovial fluid; located at MWT, about 65 KDa. (M): pre-stained protein marker; lanes 1, 2 representing samples at day 0 (control); lanes 3, 4 representing samples for the 2nd month; lane 5: samples at the 3rd month; lanes 6 and 7 samples at the 5th month; and lanes 8 and 9 corresponding to the 7th month. (**B**) Graphical representation for the level of COL2A1 protein expression level relative to total protein Coomassie blue staining) as quantified by densitometric analysis of the bands. Data presented as the mean ± SEM for triplicate samples (*n* = 3). Values with the same letters are non-significantly different (*p* > 0.05) while the values of different letters are significantly different (*p* < 0.05).

The western blotting for COL2A1protein revealed single specific bands at molecular weight ~65 KDa (Figure 6A). The expression level of COL2A1 protein was gradually increased, with a plateau expression pattern starting from the third month until the end of the experiment (*p* < 0.05) (Figure 6B).

### 3.4. Macroscopical Findings

**Figure 7:**
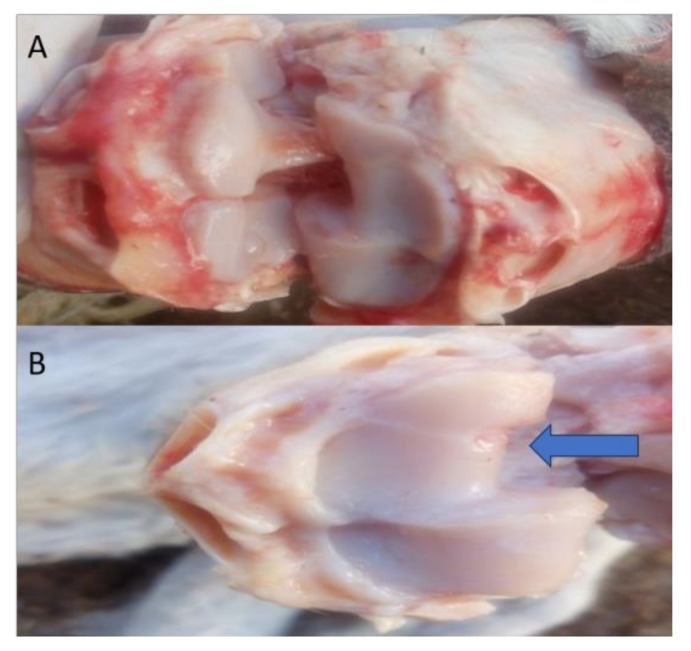
Representative images of the macroscopic observation for the distal articular surface of the radius. (**A**) Represents the right radiocarpal joint, while (**B**) Represents the left radiocarpal joint, showing erosions at the distal articular surface of the radius after the 7th month (blue arrow).

Gross macroscopic examination for the articular cartilage revealed focal erosion at the distal radial articular surface at the seventh month post-MIA induction (Figure 7).

### 3.5. Histopathological Examination

**Figure 8:**
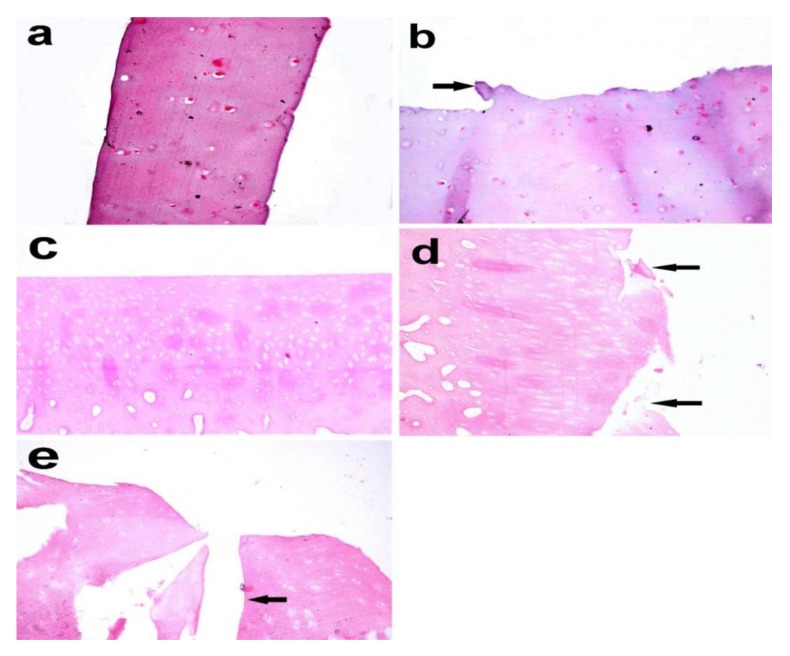
Articular cartilage biopsy of donkey. (**a**) Normal histologic features and a smooth surface in the control group (right radius articular cartilage), (**b**) uneven surface with superficial fibrillation (arrow) in theMIA-injected group (left radius articular cartilage) after 3 months (X 400). (**c**) Normal histological structure in which the matrix and chondrocytes are organized into superficial, mid, and deep zones. (**d**) Surface fibrillation and vertical branching fissures extending into mid-zone after 5 months (grade 3) (arrow). (**e**) Extensive erosion with excavation and loss of matrix in fissured domains (grade 4) after 7 months (arrow). (X 200), Haematoxylin and eosin stain.

Histopathological examination of the articular cartilage in the control group at the third month revealed normal histological features and a smooth surface (Figure 8a), whereas the surface of the articular cartilage became uneven with superficial fibrillation in the treated group (Figure 8b).In the fifth month, the articular cartilage in the control group revealed normal histological structure, in which the matrix and chondrocytes were organized into superficial, mid, and deep zones (Figure 8c). In the MIA-injected group, there was surface fibrillation and vertical branching fissures extended into the mid zone (grade 3) (Figure 8d), which became more severe at the seventh month, resulting in extensive erosion with excavation and loss of matrix in the fissured domains (grade 4) (Figure 8e).

**Figure 9:**
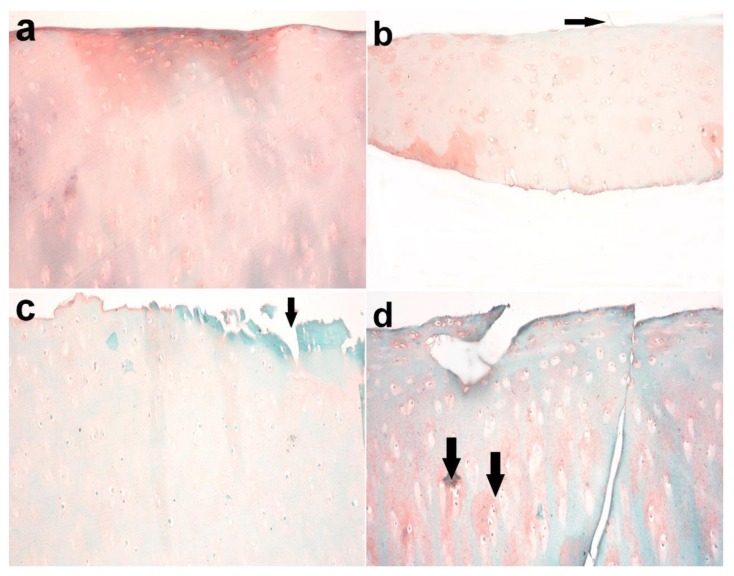
Articular cartilage of donkey. (**a**) Normal histology of articular cartilage with normally distributed chondrocytes and a smooth surface in the control group (right radius articular cartilage). (**b**) Superficial fibrillation and decreased or variable staining intensity at the surface in the MIA-injected group after 3 months (arrow). (**c**) Uneven surface and matrix fibrillation became clearer and extended vertically down into the mid-zone with the loss of proteoglycans and loss of viable chondrocytes in the treated group (arrow) after 5 months. (**d**) The fissures became more extensive and extended into the deep zone, together with further loss of proteoglycan staining and excavation after 7 months. Note the presence of clusters of chondrocyte proliferation (arrow). Safranin O stain X 200.

Using Safranin O, the articular cartilage in the control group showed normally distributed chondrocytes and a smooth surface (Figure 9a). In the MIA-injected group after the third month, on the other hand, it demonstrated superficial fibrillation and decreased or variable staining intensity at the surface (Figure 9b). At the fifth month, the articular cartilage revealed an uneven surface and matrix fibrillation became clearer and extended vertically down into the mid-zone with loss of proteoglycans and loss of viable chondrocytes (Figure 9c).In the seventh month, the fissures became more extensive and extended into the deep zone, together with further loss of proteoglycan staining and excavation. Clusters of chondrocyte proliferation were also observed (Figure 9d).

### 3.6. Immunohistochemical Analysis

**Figure 10:**
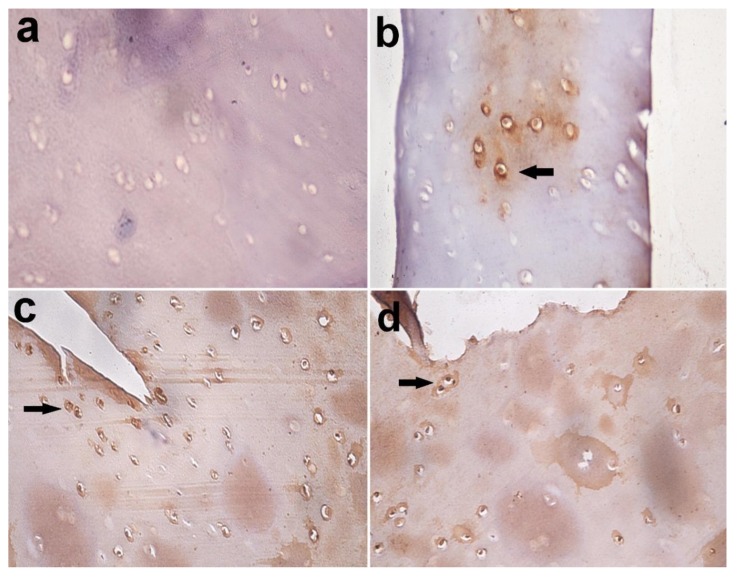
Immunohistochemical staining of Caspase-3 in articular cartilage, equine. (**a**) Caspase-3 negative chondrocytes in control group; (**b**) few Caspase-3 positive chondrocytes in the MIA-injected group after 3 months (arrow); (**c**) many Caspase-3 positive chondrocytes in the MIA-injected group after 5 months; (**d**) and in MIA-injected group after 7 months (arrow). Immuno-peroxidase stain X 400.

**Figure 11:**
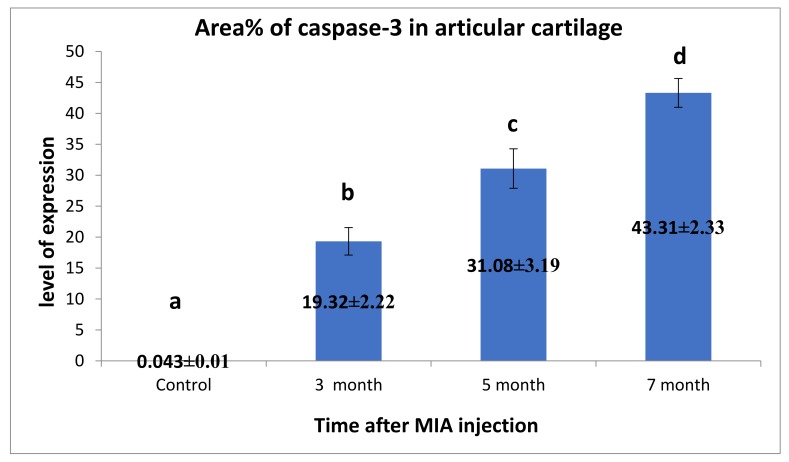
Immunohistochemical analysis for area% of Caspase-3 in examined articular cartilage revealed increased expression of Caspase-3 in chondrocytes compared to control. The expression of Caspase-3 increased with the increase of time post-MIA injection. Data presented as the mean ± SEM for triplicate samples (*n* = 3). Values with the same letters are non-significantly different (*p* > 0.05), while the values of different letters are significantly different (*p* < 0.05).

Immunohistochemical staining of Caspase-3 in the examined articular cartilage revealed increased expression of Caspase-3 in chondrocytes compared to control. The expression of Caspase-3 increased with an increase in time post-MIA injection (Figure 10 and Figure 11).

## 4. Discussion

Osteoarthritis is considered to be one of the life welfare-threatening diseases of socioeconomic burden for humans and animals [4]. Despite the great economic importance of joint diseases, especially OA in equine, studies concerning the pathophysiological mechanisms involved in joint degeneration in this species are limited [49].

Little of the literature used donkey as the OA model [32,33,34], although it is one of the equine species that is phylogenetically and biochemically closest to the horse, in addition to its low price and low cost of feedstuff and housing [34].

The current study traced the progression of clinico-histologic lesions in a chemically-induced osteoarthritic donkey model with major emphasis on MMPs and collagen metabolites.

In the present study, lameness was graded according to AAEP, where the highest score was reached after one week then decreased gradually. These results are in agreement with the previous report [34] that lameness was a major feature for MIA-injected donkeys. Also, Uilenreef et al. (2019) [37] assessed the lameness in MIA-injected pigs, where the animals showed rapid lameness which was greatly declined by the end of the experiment. In-context, MIA injection produced weight-bearing asymmetry in rats; this asymmetry was biphasic, possibly reflecting the initial inflammatory-driven phase of pain, followed by a second chronic phase that is connected to pathomorphological changes in the joint [50,51,52,53]. The observed low lameness score as the experiment proceeded in the current study could be due to the usage of a mild dose of MIA (25 mg), which did not induce severe lameness [37,54,55].

Radiographically, the gradual narrowing in the joint space, subchondral bone sclerosis, and osteophytes formation that were recorded in the current study agreed with the previous report [34]. Rat hips injected with MIA showed definite joint space narrowing and deformity of the femoral head, evidenced by a flattened epiphysis [56]. However, radiographical grading for the severity of OA in humans usually does not reflect the amount of inflammation and joint damage, therefore it does not correlate to joint pain [57,58]. These findings appeared sound to our findings; by the disease progress, the radiographic osteoarthritic changes were detected, and the lameness score decreased. Bone attrition was not observed in this study, which could be due to the dose of MIA injected, which resulted in mild to moderate osteoarthritic lesions.

Ultrasound recorded the changes in the joint cavity and articular cartilage for the first time in Egyptian donkeys in the present study. A hypo-echoic mass appeared within the synovial fluid after one month of induction and increased gradually until the third month, then decreased at the fifth and seventh months. Articular cartilage showed decreased echogenicity with a defect in its continuity in the seventh month after induction.

Intra-articular MIA injection resulted in synovial membrane hemorrhage with blood effusion, that appeared as an ultrasonographic hypoechoic mass [34]. Moreover, the hypoechoic mass could be due to edema generated by MIA injection [54]. This observed edema is due to acute inflammation, which was dependent on TRPA1(Transient Receptor Potential Ankyrin 1) activation through endogenously-formed reactive oxygen and nitrogen species and their metabolites [59,60].TRPA1 mediates its action through several mechanisms, such as mast cell degranulation, neutrophil migration, the release of histamine, serotonin, and adrenaline in concomitantwith the production of prostaglandins [61,62].Also, it enhances the expression of inflammatory genes, such as prostaglandin-producing enzyme COX-2, myeloperoxidase, and IL1β, IL-6, inducible nitric oxide synthase (iNOS), and MMPs [63,64]. Additionally, TRPA1 activation results in pain sensation, amplification of neurogenic inflammation through the release of neuropeptides such as substance P and calcitonin gene related peptide [65,66]. The reduction of inflammatory edema, pain, and lameness score with time in the current work might be due to decrement in TRPA1 expression. This hypothesis needs further study to correlate between the MIA and TRPA1 expression.

Matrix metalloproteinases have been involved in cartilage matrix degradation associated with OA [67]. Their synthesis is tightly regulated at the level of gene expression and in a tissue-specific manner [68]. Therefore, it was reasonable to assess MMPs in the synovial fluid of the joint environment [14].

Under normal physiological conditions, the expression of MMPs was low, but increased with the progression of OA, where proinflammatory cytokines such as IL-1β and TNF α are produced by chondrocytes, creating inflammatory processes, resulting in an increase in the synthesis of matrix-degrading proteinases [69].

Heparin-enhanced substrate zymography was used to evaluate the collagenases (MMP-1, MMP-13) and the gelatinases (MMP-2, MMP-9) activities in the synovial fluid for the first time in donkeys. MMP-1 and MMP-13 exhibited an undulating expression pattern, where the activity increased in the second month, then decreased and re-increased in the seventh month, also activity of MMP1 was higher than that of MMP-13. In the same line, Janusz et al. (2001) [70] reported increased activity in the collagenase and gelatinase during the first seven days after iodoacetate injection into rat knees, with a decline in activity at day 21. The level of MMP-1 expression is often 10-fold higher than the level of MMP-13 expression [71]. MMP-13 is associated with pain, disease severity, and pathogenesis of OA [72].

MMP-2 and MMP-9, in the current work, had two phases of up-regulation: peaking in the first month and between the third and seventh months, and the second peak for latent MMP-9 was between the fifth and seventh months. Elevated MMP-2 and MMP-9 activities have been detected in synovial fluid from various joint diseases in man [73,74,75], but in the equine, few data on synovial gelatinase activities are recorded [76,77,78]. Interestingly, MMP2 and MMP13 have been found in the active forms. From these data, it can be concluded that the early phase of MIA-induced OA is characterized by a decrease in the biosynthetic activity of cartilage in concomitant with the increase in catabolic activities, followed by a period of compensation where the chondrocytes try to resist the catabolic phase, which was represented in the second month, where the gelatinases activity was down-regulated.

Inline, MMP-2 expression in normal adult cartilage is weak due to very low collagen turnover, and it is upregulated in arthritis [79,80]. MMP-2 was strongly expressed in the synovial fluid of the unstable knee in the canine cruciate ligament transaction (ACLT) model of OA from the first day post-surgery, and remained high for eight months, indicating that MMP-2 is responsible for the initiation and progression of OA [81]. MMP-9 protein expressions were higher in the synovial joint fluid of patients with OA [82], and it has been demonstrated to be highly expressed in the early stages of wound healing due to its involvement in angiogenesis [83]. Pajak et al. (2017) [84] added that MMPs’ genes transcription was increased in the osteoarthritic rat model, with the highest expression in mRNA for MMP-9, which was firstly increased at day 2 then significantly decreased at day 28 compared to the gradual increase of MMP-13 to day 28. These data in concomitant with our results reveal that gelatinases initiate the degradative sequences in early OA, followed by collagenases, which play a pronounced role in OA progression. Many studies proposed MMP-2 and MMP-9 in synovial fluid as a potential biomarker for the diagnosis of OA in equine [1,85].

On the other hand, Heard et al. (2012) [14] reported that MMP levels in human knees cannot distinguish between normal and early OA stage, as MMP-1,2,9 and 13 levels appeared to be similar, while in advanced OA they seemed to be detected at a higher level.

In this report, MMP activity differentiated between normal and osteoarthritic synovial fluids, but it could not differentiate between the early and the late stages.

COL2A1 is the major component of the cartilage matrix, and its degradation by MMPs is considered a typical pathological process in OA development [86]. Qualitative immunoblot analysis in the present study recognized the COL2A1 protein as a 65 KDa common band in the synovial fluid, which is different from 140 KDa expected one. Quantitatively, our blot analysis revealed an unexpected significant increase in COL2A1 level of expression along the OA progression, starting from the second month, until the end of the study.

To our knowledge, our study is the first to monitor the expression level of COL2A1 in the synovial fluid of donkeys during the progression of OA. Regarding our surprising results, the authors postulate that the polyclonal COL2A1 antibody identified partially degraded forms of type II collagen. This assumption has been strongly raised from studies reporting that type II collagen is cleaved by collagenases (MMP-1,8 and 13), resulting in two fragments: a three-quarter-length fragment (TC^A^) and a one-quarter-length fragment (TC^B^). As a result of proteolysis, type II collagen epitopes were lost to body fluids, indicating the amount of collagen degradation [87,88,89]. Since 1989, Moreland et al. [90] has reported the presence of insoluble and partially degraded type II collagen in synovial phagocyte using a well-defined monoclonal antibody against type II collagen. The results of MMP activity where the collagenases (MMP-1 and 13)were detected in the normal synovial fluid at a low level and began to increase from the second month post-induction and the action of collagenase precede the degradation of COL2A1; this strengthens and confirms our speculation about COL2A1 appearance in the synovial fluid. Another explanation may be directed to the osteophyte formation. Osteophyte formation is a process of neochondrogenesis of mesenchymal stem cells (MSC) present in the periosteum at the bone–cartilage junction and is observed at the joint margins [91]. Osteophyte cellular differentiation has been analyzed by investigating collagen expression on mRNA and protein level [92,93]. On the mRNA level, it was found that osteophytes are composed of cells that express type I procollagen mRNA, mesenchymal prechondrocytes that express type IIA procollagen mRNA, and maturing chondrocytes that express type IIB procollagen mRNA [94]. On the protein level, Junker et al. (2015) [95] found that collagen II was strongly expressed throughout osteophyte formation. Recently, several studies stated that COL2A1 mRNA expression level was significantly increased in chondrocytes isolated from articular cartilage of high-grade OA patients and proposed that this may be a compensatory anabolic response from the chondrocytes in attempts to restore the ECM [96,97]. So, further amino acid sequence assessment of this donkey protein should be performed to confirm these speculations and define the source of collagen II.

To deduce a reliable OA grading, cartilage samples were also used for histopathological and immunohistochemical examination to ensure that synovial fluid can reflect the different status of the articular cartilage.

Histopathological findings using H&E and Safranin-O stains revealed gradual articular cartilage degeneration until it reached grade 4, according to the OARSI histopathological grading system, as well as chondrocyte proliferation. These results are in agreement with the previous studies on horse [38,98] and human OA [99]. Also, the loss of cartilage proteoglycan, as measured by Safranin O-fast green staining in MIA-induced OA in rats [71] and guinea pigs [36] is due the enzymatic activity of aggrecanase, not MMPs in cartilage cultures stimulated with IL-1. Udo et al. (2016) [100] reported that one of the mechanisms by which MIA induces arthritis is by decreasing proteoglycan content.

Chondrocytes as a sole cell present in cartilage are responsible for the maintenance of the ECM of the cartilage. During the development of OA, chondrocytes change their function and/or undergo apoptosis or chondroptosis [4]. Currently, Immunohistochemical analysis for Caspase-3 in the articular cartilage in the present work revealed a dramatic increase in the expression of Caspase-3 in chondrocyte in relation to the control samples and reached its highest level after seven months. Inline, overexpression of Caspase-3 has been noted in human OA cartilage, which correlated positively with reduced cell density and apoptosis [101].

Caspase-3 modulated chondrocyte apoptosis and destroyed the structural and biochemical homeostasis in OA [102], while the inhibition of apoptosis using caspase inhibitors reduced the severity of cartilage lesions in experimental OA [103].

MIA is a potent metabolic inhibitor, resulting in the inhibition of glyceraldehyde-3 phosphate dehydrogenase, leading to an increased level of chondrocyte apoptosis [104]. Since this enzyme is involved in the glycolysis pathway and energy production, MIA results in blocking the energy pathways and activation of the intrinsic (mitochondrial) pathway of apoptosis. The intrinsic pathway is mediated by two effectors because of MIA injection. First, by decreasing the mitochondrial energy production resulting in mitochondrial perturbation as alteration and reduction of mitochondrial membrane potential (ΔΨm), which lead to increasing the mitochondrial permeability and release of pro-apoptotic factor (cytochrome-c), which results in activation of Caspase-3. The second one through the increased production of ROS, which reduces mitochondrial ΔΨm, and finally both effectors combine and result in the activation of Caspase-3 and apoptosis initiation. Caspase-3 promotes the typical apoptosis features, including DNA fragmentation and cell death, in many tissues, including cartilage [105]. From the obtained results, COL2A1 and Caspase-3 can be considered promising prognostic biomarkers for OA development. Also, they can give an idea about the proper timing for traditional therapeutic intervention by MMP inhibitors, like tetracycline derivatives, which will help in improving the prognosis and reducing the economic losses resulting from such disease.

## 5. Conclusions

Although our study had a limitation concerning the small number of animals used in attempts for the establishment of grading diagnostic biomarkers for the first time in Egyptian donkeys, it would be helpful to extend further work on that socioeconomic disease. The low dose of MIA (25 mg/mL) showed the features of OA in donkeys in a time-dependent manner (seven months) and enabled us to follow up and grade the changes for the first time in donkeys. Based on different parameters included in the study, clinical, radiographical, and ultrasonographic examination with MMPs activity, COL2A1 protein expression level, histopathological and immunohistochemical findings, we assumed that OA stages could be graded into: 1—the very early stage (the first month) and characterized by increased the expression of gelatinases; 2—early-stage (represented in the second month) and characterized by the maximum activity of collagenases (MMP-1 and MMP-13) with non-detected activity for gelatinases; 3—late-stage, starting from the third month, characterized by enhanced activity of all MMPs. Accordingly, therapeutic intervention until the end of the early stage could represent a good prognosis in relation to the levels of COL2A1 and Caspase-3.

Despite the fine regulation for the activity of MMPs, they cannot differentiate between the early and late stages of osteoarthritis and we cannot depend on them as an early diagnostic biomarker. So, our group extended the research to precisely define diagnostic, prognostic early biomarkers depending on the circulating miRNAs and gene-controlled metabolic pathways involved in MIA-induced OA in donkeys.

## Figures and Tables

**Table 1:** Lameness score according to American Association of Equine Practitioners (AAEP).

Experimental Periods	Zero Day	1st Week	1st Month	2nd Month	3rd Month	5th Month	7th Month
Lameness score	0	3rd degree	2nd degree	1st degree	1st degree	1st degree	1st degree

**Table 2:** Osteoarthritis Research Society International (OARSI) grading scores according to analysis of dorso-palmar radiographs.

OARSI	Zero Day	1st Week	1st Month	2nd Month	3rd Month	5th Month	7th Month
Marginal osteophyte	0	0	0	0	0	1	1
Joint space narrowing	0	0	1	2	2	3	3
Sub-chondral sclerosis	Absent	Absent	Absent	Absent	Present	Present	Present
Bone attrition	Absent	Absent	Absent	Absent	Absent	Absent	Absent
